# Screening of natural *Wolbachia* infection in mosquitoes (Diptera: Culicidae) from the Cape Verde islands

**DOI:** 10.1186/s13071-023-05745-w

**Published:** 2023-04-25

**Authors:** Aires Januário Fernandes da Moura, Vera Valadas, Silvania Da Veiga Leal, Eddyson Montalvo Sabino, Carla A. Sousa, João Pinto

**Affiliations:** 1 Global Health and Tropical Medicine, GHTM, Instituto de Higiene e Medicina Tropical, IHMT, Universidade Nova de Lisboa, UNL., Rua da Junqueira 100, 1349-008, Lisboa, Portugal; 2 Unidade de Ciências da Natureza, da Vida E Do Ambiente, Universidade Jean Piaget de Cabo Verde, Praia, Cape Verde; 3 Laboratório de Entomologia Médica, Instituto Nacional de Saúde Pública, Praia, Cape Verde; 4 Laboratório de Simulidos, Universidad Nacional Hermilio Valdizan, Huánuco, Peru

**Keywords:** *Wolbachia*, Genotyping, Mosquitoes, *Culex pipiens*, *Culex tigripes*, Cape Verde

## Abstract

**Background:**

*Wolbachia pipientis* is an endosymbiont bacterium that induces cytoplasmic incompatibility and inhibits arboviral replication in mosquitoes. This study aimed to assess *Wolbachia* prevalence and genetic diversity in different mosquito species from Cape Verde.

**Methods:**

Mosquitoes were collected on six islands of Cape Verde and identified to species using morphological keys and PCR-based assays. *Wolbachia* was detected by amplifying a fragment of the surface protein gene (*wsp*). Multilocus sequence typing (MLST) was performed with five housekeeping genes (*coxA*, *gatB*, *ftsZ*, *hcpA*, and *fbpA*) and the *wsp* hypervariable region (HVR) for strain identification. Identification of *w*Pip groups (*w*Pip-I to *w*Pip-V) was performed using PCR–restriction fragment length polymorphism (RFLP) assay on the ankyrin domain gene *pk1*.

**Results:**

Nine mosquito species were collected, including the major vectors *Aedes aegypti*, *Anopheles arabiensis*, *Culex pipiens *sensu stricto, and *Culex quinquefasciatus*. *Wolbachia* was only detected in *Cx. pipiens *s.s. (100% prevalence), *Cx. quinquefasciatus* (98.3%), *Cx. pipiens*/*quinquefasciatus* hybrids (100%), and *Culex tigripes* (100%). Based on the results of MLST and *wsp* hypervariable region typing, *Wolbachia* from the *Cx. pipiens* complex was assigned to sequence type 9, *w*Pip clade, and supergroup B. PCR/RFLP analysis revealed three *w*Pip groups in Cape Verde, namely *w*Pip-II, *w*Pip-III, and *w*Pip-IV. *w*Pip-IV was the most prevalent, while *w*Pip-II and *w*Pip-III were found only on Maio and Fogo islands. *Wolbachia* detected in *Cx. tigripes* belongs to supergroup B, with no attributed MLST profile, indicating a new strain of *Wolbachia* in this mosquito species.

**Conclusions:**

A high prevalence and diversity of *Wolbachia* was found in species from the *Cx. pipiens* complex. This diversity may be related to the mosquito's colonization history on the Cape Verde islands. To the best of our knowledge, this is the first study to detect *Wolbachia* in *Cx. tigripes*, which may provide an additional opportunity for biocontrol initiatives.

**Graphical Abstract:**

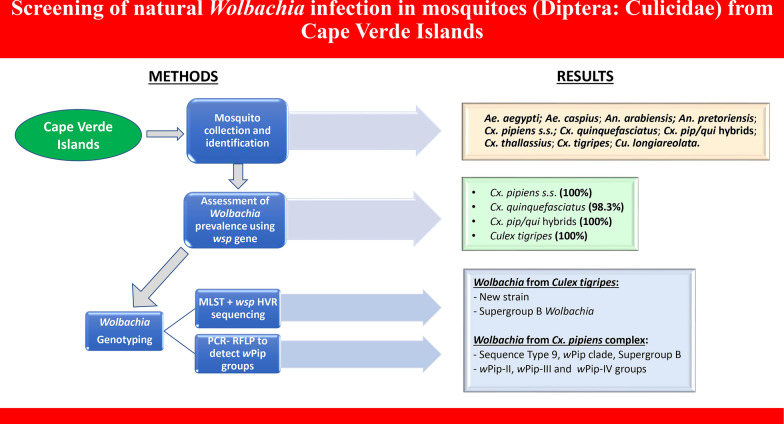

**Supplementary Information:**

The online version contains supplementary material available at 10.1186/s13071-023-05745-w.

## Background

*Wolbachia pipientis* (Alphaproteobacteria, *Rickettsiales*) is an obligate intracellular gram-negative bacterium and proteobacterial symbiont found in a variety of invertebrates, including insects, crustaceans, arachnids, and filarial nematodes [1]. Currently, the *Wolbachia* genus is subdivided into 17 supergroups (A–F; H–Q, and S), and most species known belong to supergroups A and B [2].

*Wolbachia* is transmitted vertically through host eggs and can influence longevity and reproduction, including feminization, parthenogenesis, and incompatibility between the female and male sex cells [3]. The best-known phenotype induced by *Wolbachia* in arthropods is cytoplasmic incompatibility (CI). It occurs when males harboring *Wolbachia* are crossed with uninfected females or between individuals infected with incompatible strains [4, 5]. The generally accepted model stipulates that cytoplasmic incompatibility results from a *Wolbachia* “modification” factor (mod; toxin) in the sperm that blocks early embryogenesis, and a *Wolbachia* “rescue” factor (resc; antitoxin) produced in the oocyte that allows the diploid zygote to develop if the cross is compatible [6, 7].

Besides cytoplasmic incompatibility, *Wolbachia* can inhibit viral replication in mosquitoes, including Zika, dengue, West Nile, and chikungunya arboviruses in *Aedes aegypti* [8, 9]. Other studies also suggest inhibition of pathogens such as *Plasmodium falciparum* in *Anopheles stephensi* and *Anopheles gambiae* and West Nile virus in *Culex quinquefasciatus* [1, 10, 11]. These abilities make *Wolbachia* a promising tool against mosquito-borne diseases and possibly an alternative to conventional vector control programs using insecticides. In fact, the release of males harboring incompatible *Wolbachia* into target populations has successfully decreased reproduction by sterilization [12, 13]. The release of *Ae. aegypti* transfected with the *Wolbachia w*Mel strain (derived from *Drosophila melanogaster*) led to the establishment of *Ae. aegypti* populations infected with *Wolbachia* and a proven decrease in dengue incidence in Australia [14] and Malaysia [15].

Cape Verde is threatened by several species of vector mosquitoes, including *Ae. aegypti*, *Anopheles arabiensis*, *Cx. quinquefasciatus*, and *Culex pipiens *sensu stricto (s.s.) [16]. Integrated vector control strategies are mainly directed against *An. arabiensis* and *Ae. aegypti,* using chemical insecticides, diesel, and biological control with *Gambusia* sp. fish [17]. However, despite control efforts, the country had its first dengue epidemic in 2009, followed by an outbreak of Zika in 2015–2016 [18] and a malaria outbreak in 2017 [19].

There is no data on the genetic diversity of *Wolbachia* infecting mosquitoes (Diptera: Culicidae) from the Cape Verde islands. This knowledge would be a first step for the design and implementation of programs to suppress mosquito populations through cytoplasmic incompatibility. In this context, the present study aims to detect and genetically characterize *Wolbachia* in populations of Culicidae from Cape Verde.

## Methods

### Study area and sample collection

**Fig. 1:**
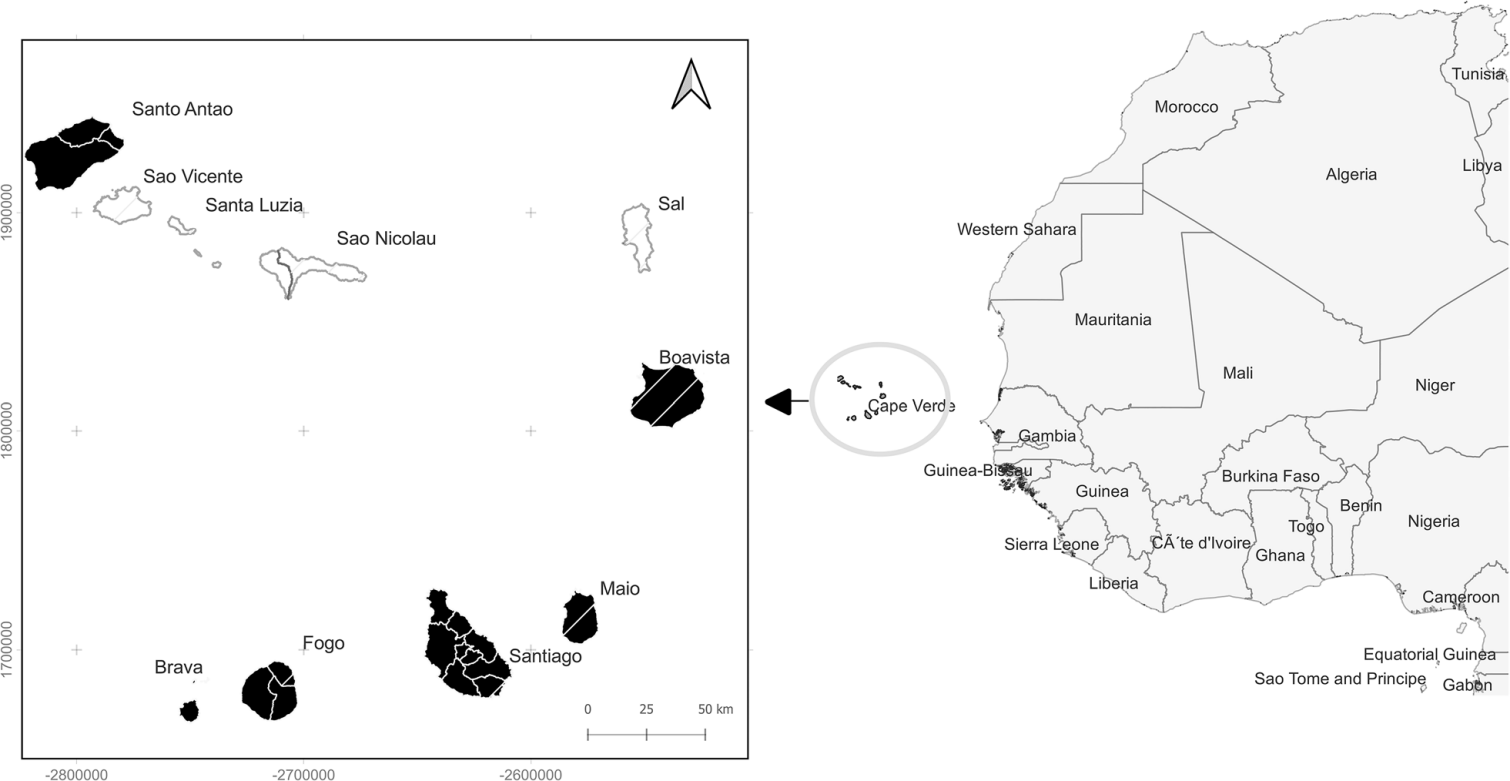
Map of the North Atlantic region showing the geographic location of the Cape Verde islands. Mosquito samples were collected on the islands of Santo Antão, Boavista, Maio, Santiago, Fogo, and Brava (highlighted in black)

An entomological survey was carried out in Cape Verde between February and June 2021. Larval and adult mosquito samples were collected on six islands (Santiago, Brava, Fogo, Maio, Santo Antão, and Boavista; Fig. 1) using BG-Sentinel and Centers for Disease Control and Prevention (CDC) light traps, dorsal aspirators, dippers, and pipettes. All collection sites were geo-referenced with a portable global positioning system (GPS) device (Garmin eTrex 10).

Mosquitoes were identified to species/complex using the Ribeiro et al. [20] identification key and stored individually in microtubes containing silica gel (for adults) or 80% ethanol (for larvae). For genetic analysis, DNA was extracted from single specimens using cetrimonium bromide (CTAB) 2% and proteinase K, according to Weeks et al. [21].

Species of the *An. gambiae* complex were identified by polymerase chain reaction (PCR) according to Scott et al. [22] using primer sequences described in Table S1 (Additional file 1: Table S1). PCR was performed using 12.5 µl of Xpert Taq^PLUS^ Mastermix (GriSP), 0.1 µM of ME and UN primers, 0.05 µM of GA primer, and 0.15 µM of AR primer, plus 1 µl of DNA template and water to a final volume of 25 µl. Cycling conditions were as follows: one cycle at 95 °C for 5 min, 30 cycles at 94 °C for 30 s, 50 °C for 30 s, and 72 °C for 30 s; and a final cycle of 72 °C for 5 min.

For the *Cx. pipiens* complex, specimens were identified to species by PCR amplification of acetylcholinesterase-2 (*ace-2*) gene sequences using primers described by Smith & Fonseca [23] (Additional file 1: Table S1). PCR was performed using 12.5 µl of Xpert Taq^PLUS^ Mastermix (GriSP), 0.4 µM of ACEquin and B1246 primer, 0.2 µM of ACEpip primer, 1 µl of DNA template and water to a final volume of 25 µl. Cycling conditions were performed as follows: one cycle at 94 °C for 5 min, 35 cycles at 94 °C for 30 s, 55 °C for 30 s, 72 °C for 1 min, and one cycle at 72 °C for 5 min.

Whenever necessary, morphological identification of species other than the above was supported with the sequencing of a 710-base-pair (bp) fragment of cytochrome c oxidase subunit 1 mitochondrial gene (*COI*) with primers LCOI1490_F1 and HCOI2198_R1 (Additional file 1: Table S1) according to Folmer et al. [24]. PCR was performed using 1X PCR buffer, 2 mM MgCl2, 0.2 mM dNTPs, 1 U Taq polymerase (Robust HotStart PCR Kit, Roche/Kapa Biosystems), 0.5 µM of each primer, 2 µl of DNA template, and water to a final volume of 20 µl. Cycling conditions were as follows: initial denaturation at 94 °C for 3 min; 40 cycles at 94 °C for 50 s; annealing at 45 °C during 30 s and 72 °C for 1 min; and final elongation at 72 °C for 5 min.

### Screening of *Wolbachia*

*Wolbachia* detection in mosquito samples was performed by amplifying a 610-bp region of the *Wolbachia* surface protein gene (*wsp*) using primers 81F and 691R (Additional file 1: Table S2) described by Zhou et al. [25]. The amplification reaction comprised 12.5 µl of Xpert Taq^PLUS^ Mastermix (GriSP), 0.4 µM of each primer, 1 µl of DNA template, and water to a final volume of 25 µl. Cycling conditions were as follows: one cycle at 95 °C for 3 min, 35 cycles at 95 °C for 1 min, 55 °C for 1 min, 72 °C for 1 min, and one cycle at 72 °C for 10 min.

All PCR products from the assays described above were analyzed by electrophoresis on a 1.5% agarose gel stained with GreenSafe Premium (NZYTech).

#### *Wolbachia* multilocus sequence typing (MLST) and *wsp* typing

*Wolbachia* genotyping was performed through amplification and sequencing of five MLST loci (*gatB*, *coxA*, *hcpA*, *ftsZ*, *fbpA*) and the *wsp* hypervariable region [26, 27]. The primer pairs for each locus and the size of amplified products are shown in supplemental materials (Additional file 1: Table S3).

PCR for each locus was performed using 1X PCR buffer, 0.2 mM dNTPs, 1.5 mM MgCl2, 0.5U Taq polymerase (Robust HotStart PCR Kit, Roche/Kapa Biosystems), 1 µM of each primer, 2 µl of DNA template, and water to a final volume of 40 µl. Cycling conditions were as follows: initial denaturation at 94 °C for 2 min; 37 cycles at 94 °C for 30 s, annealing at 54 °C (for *hcpA*, *gatB*, *ftsZ*, and *coxA*), and 59 °C (*fbpA* and *wsp*) for 45 s, and 72 °C for 90 s; and final elongation at 72 °C for 10 min.

Five microliters of PCR product from each locus was used in electrophoresis to confirm amplification. The remaining 35 µl was purified using an Exo/SAP Go PCR purification kit (GriSP) and sent for direct DNA sequencing at STAB Vida (Oeiras, Portugal) using forward and reverse primers.

*Wolbachia* MLST and hypervariable *wsp* sequences were edited and aligned using BioEdit (version 7.0.9.0). Consensus and concatenated sequences (*gatB*, *coxA*, *fbpA*, *ftsZ*, *hcpA*, and *wsp* hypervariable region [HVR]) were queried in the *Wolbachia* MLST database (https://pubmlst.org/bigsdb?db=pubmlst_wolbachia_seqdef) for strain characterization. Sequences were also subjected to the nucleotide Basic Local Alignment Search Tool (BLAST) to verify the similarity with deposited sequences in GenBank (https://blast.ncbi.nlm.nih.gov/Blast.cgi).

Phylogenetic analysis was conducted using the gamma-distributed Tamura 3-parameter nucleotide substitution model, and a neighbor-joining tree was generated employing 1000 bootstraps in Molecular Evolutionary Genetics Analysis version 11 (MEGA11) [28].

#### Identification of *w*Pip groups by PCR–RFLP

Identification of *w*Pip groups (*w*Pip-I to *w*Pip-V) was performed using a PCR–restriction fragment length polymorphism (RFLP) assay based on the ankyrin (ANK) *Wolbachia* marker *pk1* [29–31]. A PCR that amplifies a 1300-bp fragment of the ANK domain gene (*pk1*) was performed with primers pk1_For and pk1_Rev (Additional file 1: Table S2) [32]. The reaction components included 10 µl of Xpert Taq^PLUS^ Mastermix (GriSP), 0.4 µM of each primer, 2 µl of DNA template, and water to a final volume of 20 µl. Cycling conditions were as follows: one cycle at 94ºC for 5 min; 35 cycles at 94 °C for 30 s, 52ºC for 30 s, and 72 °C for 90 s; and a final cycle of 72 °C for 5 min. PCR product was analyzed by electrophoresis on a 2% agarose gel stained with GreenSafe Premium (NZYTech).

The *pk1* PCR product was digested with restriction enzymes *Taq*αI and *Pst*I to identify different *w*Pip groups [29]. Digestion with *Taq*αI was performed with the following reaction mixture: 2 µl of Buffer C (NZYTech), 10 µl of the PCR product, 18 µl of water, and 2 µl *Taq*αI enzyme (NZYTech) at 10U/µl. The mixture was placed in a thermal cycler at 65ºC for 90 min. The reaction was stopped by adding 0.02 mM of ethylenediaminetetraacetic acid (EDTA) (pH = 8) to each tube, and the digestion product was visualized by electrophoresis on a 2% agarose gel. Each allele (*w*Pip group) was detected according to the size of the resulting fragments: allele “a” or “e” (*w*Pip-I or *w*Pip-V; 991, 251, 107 bp); “b” (*w*Pip-III; 669, 665 bp); “c” (*w*Pip-II; 851, 498 bp); “d” (*w*Pip-IV; 497, 251, 107 bp) [29].

If alleles "a" or "e" (*w*Pip-I or *w*Pip-V) were present, the two were differentiated by digesting the *pk1* PCR product with the *Pst*I restriction enzyme. For this purpose, a reaction mixture was prepared with 2 µl of Buffer A (NZYTech), 12 µl pk1 PCR product, 1 µl *Pst*I enzyme (NZYTech) at 10U/µl, and 5 µl of water. The mixture was incubated at 37 °C for 1 h, and the reaction stopped by incubating at 80 °C for 20 min. Digested DNA fragments were separated by electrophoresis on a 2% agarose gel. *w*Pip alleles resulting from *pstI* digestion included “a” (*w*Pip- I; 903, 303, 141 bp) and “e” (*w*Pip-V; 903, 430 bp) [29, 30].

Sequencing of *pk1* PCR products was performed to confirm the RFLP profile. For this purpose, the *pk1* PCR product was purified as described above for the MLST and sent for direct sequencing using reverse and forward primers. Sequences were subjected to the nucleotide BLAST, and phylogenetic analysis was performed using the gamma-distributed Tamura 3-parameter nucleotide substitution model, and a neighbor-joining tree was generated using 1000 bootstraps in MEGA software version 11.0.11.

## Results

### Mosquito species identification

A total of 1648 mosquitoes (303 larvae and 1345 adults) were collected (Additional file 2: Table S4 for details). Species identification by morphological characters revealed the presence of *Ae. aegypti* (*n* = 663, 40.2%), *Aedes caspius* (*n* = 39, 2.4%)*, An. gambiae *sensu lato (s.l.) (*n* = 49, 3.0%)*, Anopheles pretoriensis* (*n* = 275, 16.7%), *Cx. pipiens *s.l. (*n* = 584, 35.4%), *Culex thalassius* (*n* = 7, 0.4%), *Culex tigripes* (*n* = 3, 0.2%), and *Culiseta longiareolata* (*n* = 28, 1.7%).

Ribosomal DNA PCR for identifying species of the *An. gambiae* complex revealed that all collected specimens from this complex belonged to *An. arabiensis*. For the *Cx. pipiens* complex, specimens were identified by *ace-2* PCR as *Cx. pipiens* s.s. (*n* = 10, 1.7%), *Cx. quinquefasciatus* (*n* = 545, 93.3%), and *Cx. pipiens/Cx. quinquefasciatus* hybrids (*n* = 29, 5.0%).

### Screening of *Wolbachia*

The *wsp* fragment was amplified only in *Cx. pipiens* s.s. (10/10 = 100% prevalence), *Cx. quinquefasciatus* (536/545 = 98.3%), *Cx. pipiens/Cx. quinquefasciatus* hybrids (29/29 = 100%), and *Cx. tigripes* (3/3 = 100%). The remaining species were negative for *Wolbachia.*

### *Wolbachia* MLST and *wsp* typing

**Fig. 2:**
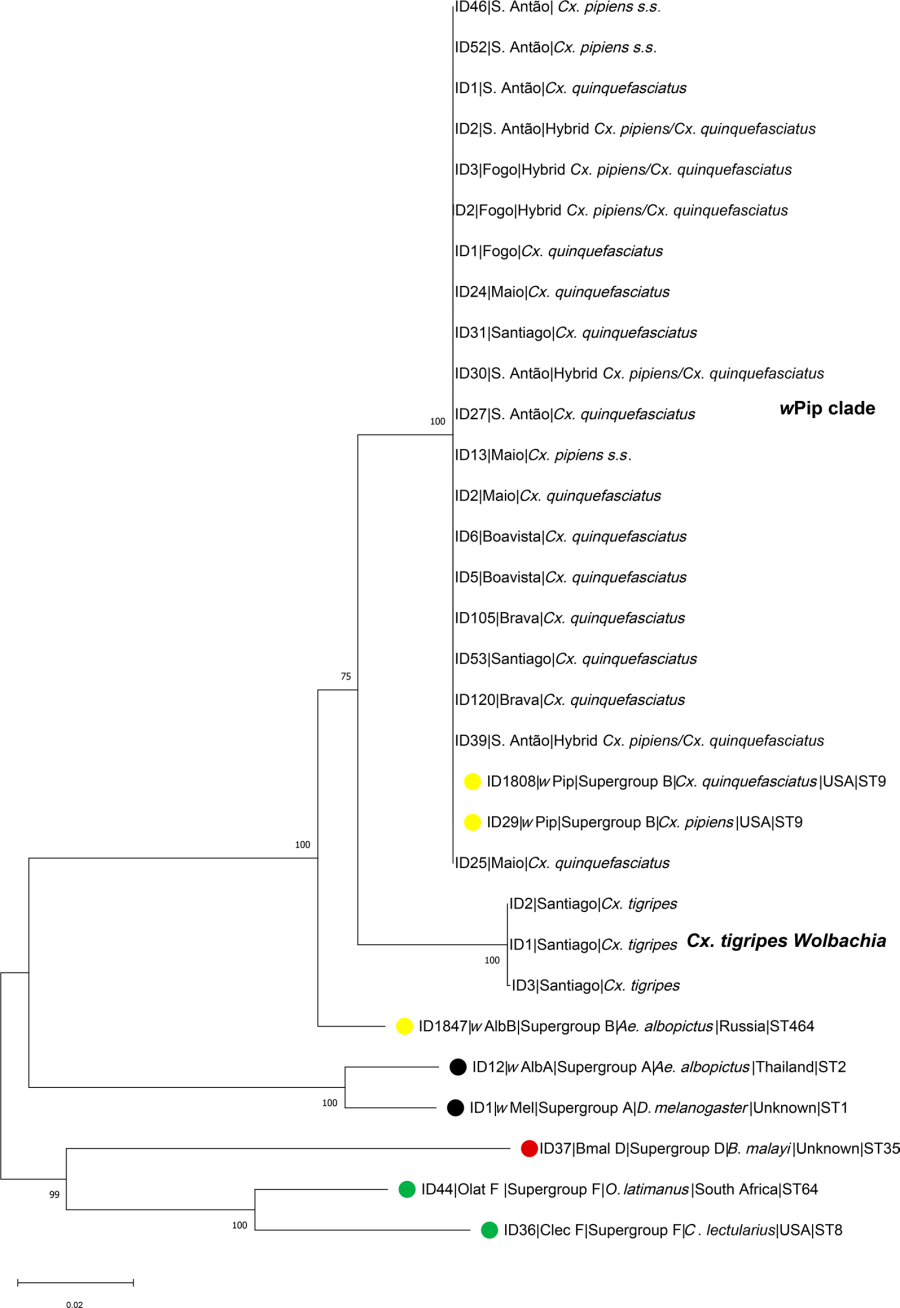
Phylogenetic tree generated from concatenated sequences of MLST loci (*coxA*, *gatB*, *ftsZ*, *fbpA*, *hcpA*) and the wsp hypervariable region. Numbers on branches indicate percentage bootstrap support (1000 replicates). Reference sequences were obtained from the *Wolbachia* MLST database and are marked by full circles. Each *Wolbachia* supergroup is marked with a different color: yellow, supergroup B; black, supergroup A; red, supergroup D; and green, supergroup F. The scale bar indicates the number of substitutions

We analyzed 80 mosquitoes that were positive for *wsp* for *Wolbachia* MLST and *wsp* typing*.* Allelic profiles resulting from MLST loci and the *wsp* hypervariable region sequencing revealed that *Wolbachia* from *Cx. pipiens* s.s., *Cx. quinquefasciatus*, and *Cx. pipiens/Cx. quinquefasciatus* hybrids belong to sequence type 9, *w*Pip clade, and supergroup B *Wolbachia* (Table 1). The same result was obtained from phylogenetic analysis using concatenated sequences of MLST loci (*coxA*, *gatB*, *ftsZ*, *fbpA*, *hcpA*) and the *wsp* hypervariable region (Fig. 2).

**Table 1:** Allelic profile of MLST genes and *wsp* hypervariable region for different species of Culicidae collected in Cape Verde islands

Host species	Island (*n*)	*gatB*	*coxA*	*hcpA*	*ftsZ*	*fbpA*	*wsp*	*HVR1*	*HVR2*	*HVR3*	*HVR4*	*ST*
*Cx. quinquefasciatus*	Santiago (*n* = 15) Brava (*n* = 15) Boavista (*n* = 12) Maio (*n* = 7) Fogo (*n* = 1) S. Antão (*n* = 10)	4	3	3	22	4	10	10	8	10	8	9
*Cx. pipiens s.s.*	Maio (*n* = 2) S. Antão (*n* = 2)	4	3	3	22	4	10	10	8	10	8	9
*Hybrids pip/qui*	Maio (*n* = 1) Fogo (*n* = 2) S. Antão (*n* = 10)	4	3	3	22	4	10	10	8	10	8	9
*Cx. tigripes*	Santiago (*n* = 3)	9	182^b^	12	117	203^b^	NA^a^	NA^a^	232	222	84	NA^a^

^a^ NA represents allelic profile or sequence type not available in the *Wolbachia* MLST database

^b^ Sequences with partial match in the *Wolbachia* MLST database

For *Cx. tigripes*, the allelic profile obtained was unavailable in the MLST database, thus not allowing the determination of a sequence type. However, the phylogenetic analysis indicates that *Wolbachia* from *Cx. tigripes* also belongs to supergroup B but integrates a distinct clade from *w*Pip (Fig. 2)*.*

### *w*Pip groups and their distribution in the archipelago

Results from *pk1* PCR–RFLP showed the occurrence of three different *w*Pip groups in Cape Verde, namely *w*Pip-IV (88.9%), *w*Pip-II (7.4%), and *w*Pip-III (3.7%) (Table 2). The *w*Pip-IV group was detected in *Cx. quinquefasciatus* from five islands (Santiago, Brava, Santo Antão, Maio and Boavista) and in *Cx. pipiens* s.s. from Santo Antão. The *w*Pip-II group was detected only in *Cx. pipiens* s.s. from Maio, while *w*Pip-III was found exclusively in *Cx. quinquefasciatus* and *Cx. pipiens*/*quinquefasciatus* hybrids from the island of Fogo (Table 2).

**Table 2:** *w*Pip groups detected in *Cx. pipiens* s.l. from Cape Verde islands according to *pk1* PCR–RFLP

Islands	Species	*n*	*w*Pip group
Santiago	*Cx. quinquefasciatus*	15	*w*Pip- IV
Brava	*Cx. quinquefasciatus*	15	*w*Pip- IV
Santo Antão	*Cx. pipiens*	2	*w*Pip- IV
	*Cx. quinquefasciatus*	10	*w*Pip- IV
	Hybrids *Cx. pipiens/quinquefasciatus*	10	*w*Pip- IV
Maio	*Cx. pipiens*	6	*w*Pip- II
	*Cx. quinquefasciatus*	7	*w*Pip- IV
	Hybrids *Cx. pipiens/quinquefasciatus*	1	*w*Pip- IV
Fogo	*Cx. quinquefasciatus*	1	*w*Pip- III
	Hybrids *Cx. pipiens/quinquefasciatus*	2	*w*Pip- III
Boavista	*Cx. quinquefasciatus*	12	*w*Pip- IV

**Fig. 3:**
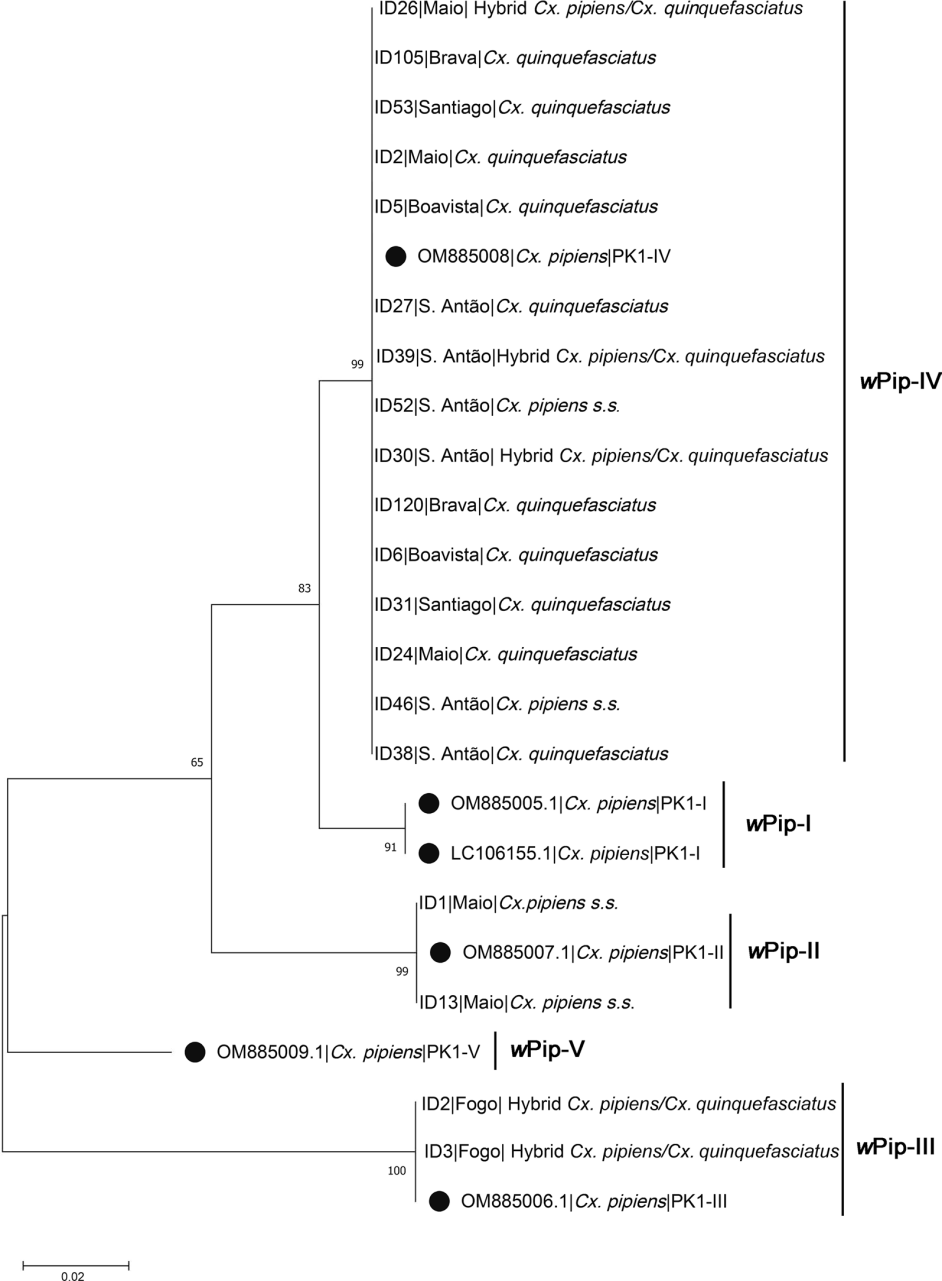
Phylogenetic tree generated from pk1 sequences by Bayesian analysis. Known *w*Pip group pk1 sequences are marked by full circles. Numbers on branches indicate percentage bootstrap support (1000 replicates). The scale bar indicates the number of substitutions

Sequencing of *pk1* PCR products confirmed the observed RFLP profiles and similarity with *pk1* sequences deposited in GenBank (Fig. 3).

## Discussion

*Wolbachia* has garnered substantial attention for its ability to control diseases transmitted by mosquitoes. This study represents the first assessment of *Wolbachia*'s prevalence and genetic diversity in mosquitoes from Cape Verde. Our objective is to expand knowledge of this bacterium through our findings and illustrate its potential for controlling mosquito-borne diseases in the archipelago.

The MLST and *wsp* typing results revealed that *Wolbachia* from *Cx. pipiens* and *Cx. quinquefasciatus* and their hybrids belong to the *w*Pip clade and share a monophyletic origin within *Wolbachia* group B. The same results were obtained by Atyame et al. [33] and Dumas et al. [30] when studying *Wolbachia* genetic diversity from Cx. *pipiens* s.l. populations originating from different regions of the world. According to the authors, these findings suggest that *w*Pip strains comprise a recent clade of the *Wolbachia* supergroup B [30, 33].

The analysis of the fast-evolving *pk1* gene revealed further variation within the *w*Pip strain, indicating the presence of *w*Pip-II, *w*Pip-III, and *w*Pip-IV groups in Cape Verde. The occurrence of different *w*Pip groups suggests multiple introduction events into the archipelago. In the past, Cape Verde was a maritime hub between Europe and mainland Africa, and the intense movement of ships may explain the diversity of *w*Pip found on the islands. This result contrasts with that of the southwestern Indian Ocean islands, in which *Wolbachia* infecting *Cx. quinquefasciatus* all belonged to the *w*Pip-I group [13]. It is noteworthy that *w*Pip-I was the only group found in mainland Sub-Saharan Africa, South America, and Southeast Asia, whereas only *w*Pip-III was detected in North America [30, 33]. Europe shows the highest diversity, with all five groups of the *w*Pip clade being found in this continent [30]. The presence of *w*Pip-II, *w*Pip-III, and *w*Pip-IV groups in Cape Verde islands suggests at least three introduction events of *Wolbachia* possibly originating from Europe. However, a North American origin for the *w*Pip-III group in Fogo Island cannot be excluded. Interestingly, the differences found in the genetic composition of the *w*Pip clade among islands agree with the genetic structure of the *Cx. pipiens* complex in Cape Verde. Previous microsatellite-based analysis suggested that *Cx. quinquefasciatus* from Fogo Island may comprise a genetic ancestry cluster distinct from the other islands [34]. What was previously considered an admixed *Cx. quinquefasciatus* population in Fogo Island [34] may, in fact, represent a genetically differentiated population originating from a *w*Pip-III group source population.

The absence of the African *w*Pip-I group from Cape Verde *Cx. quinquefasciatus* is not easily explained. Mainland Africa would be the natural candidate for a source population of *w*Pip-I *Cx. quinquefasciatus* that would have colonized the Cape Verdean islands, as suggested for the southwestern Indian Ocean islands [30]. However, *Cx. quinquefasciatus* was predominantly infected by the *w*Pip-IV group. This result may suggest that *Cx. quinquefasciatus* from Cape Verde may have derived from a yet to be sampled *w*Pip-IV population of mainland Africa. Another explanation would involve the cytoplasmic transfer of *w*Pip-IV from European *Cx. pipiens* s.s. to *w*Pip-I *Cx. quinquefasciatus* via hybridization, followed by the latter's replacement through cytoplasmic incompatibility (CI). High levels of CI have been reported in crosses between *w*Pip-II and *w*Pip-IV, as well as between *w*Pip-III- and *w*Pip-IV-infected mosquitoes [7, 29]. Studies involving experimental crosses would be required to assess CI between *w*Pip-I and *w*Pip-IV and whether this CI would confer an adaptive advantage to *w*Pip-IV-infected mosquitoes.

*Wolbachia* was not detected in *Ae. aegypti* from Cape Verde islands, which is consistent with most surveys on this species where no evidence of *Wolbachia* natural infection was found [35–38]. The presence of *Wolbachia* in *Ae. aegypti* has been reported on only a few occasions, including those from New Mexico, the USA [39], and Kuala Lumpur, Malaysia [40]. However, the possibility of *Wolbachia* detection in *Ae. aegypti* being the result of an infection with a *Wolbachia*-carrying nematode or of environmental contamination during field collections could not be excluded [36]. *Wolbachia* was also not detected in *An. arabiensis* and *An. pretoriensis* from Cape Verde. While this result is in line with most studies that screened for *Wolbachia* in *Anopheles* species [41, 42], there have been a few reports on the presence of the endosymbiont in *An. gambiae* and *An. coluzzii* from Mali [43], *An. gambiae* from the Democratic Republic of Congo, and *An. coluzzii* in Ghana [44]. Shaw et al. [45] concluded that *Wolbachia* natural *Anopheles* infections do not induce cytoplasmic incompatibility or sex ratio distortion but show a negative correlation with *Plasmodium* infection, suggesting that *Wolbachia* may interfere with malaria transmission.

This study reports for the first time the presence of *Wolbachia* in *Cx. tigripes*. Phylogenetic analyses indicate that *Wolbachia* isolated from this mosquito belongs to supergroup B, with no attributed MLST profile. This result suggests the presence of a new strain of *Wolbachia* infecting *Cx. tigripes* in Santiago Island. *Culex tigripes* is the only predatory mosquito in Cape Verde [46], and on the island of Santiago, its larvae are often found in breeding sites associated with *Cx. pipiens* s.l. species [47]. Our results exclude environmental contamination by *Cx. pipiens* s.l. *Wolbachia* since we detected *Wolbachia* in both larvae and an adult male of *Cx. tigripes* (Additional file 2: Table S4). More importantly, the concatenated sequences of the MLST loci and the *wsp* HVR region clearly showed that the strain detected in *Cx. tigripes* forms a monophyletic group separate from the *w*Pip clade. Our phylogenetic analyses also exclude contamination with *Wolbachia* from supergroups D and F, which are generally found in nematodes [2, 48].

The use of *Wolbachia*-based methods in vector management holds significant promise. The newly detected *Wolbachia* strain in *Cx. tigripes* from Cape Verde encourages further research to assess their ability to be firmly established in major vector trans-infected lines, induce cytoplasmic incompatibility, or reduce the ability to transmit pathogens. Proof of these abilities may offer an additional opportunity for biocontrol initiatives.

The incompatible insect technique (IIT), a variation of the sterile insect technique (SIT), can be performed by taking advantage of the *w*Pip-induced cytoplasmic incompatibility. Studies have indicated that *Cx. pipiens* s.l. mosquitoes infected with identical *Wolbachia w*Pip groups tend to exhibit cytoplasmic compatibility, while crossing between mosquitoes carrying different *w*Pip groups is often incompatible [13, 31]. As a result, our findings regarding the natural occurrence of *w*Pip groups in Cape Verde can provide valuable insights for implementing control programs for *Cx. pipiens* s.l. in the archipelago.

Experiments conducted in semi-field conditions on La Réunion showed that the mating between local *Cx. quinquefasciatus w*Pip-I females and non-native males carrying the *w*Pip-IV (Istanbul strain) resulted in 100% embryonic mortality [13]. Altinli et al. [29] demonstrated naturally occurring CI patterns between *w*Pip-IV-harboring males and *w*Pip-I- or *w*Pip-II-harboring females in *Cx. pipiens* s.l. populations from Turkey. These observations reveal that IIT based on *w*Pip-inducing IC could be employed to control *Cx. pipiens* populations. The same methodology can be implemented in Cape Verde considering the data we gathered on the prevalence and distribution of *w*Pip groups in the archipelago. It would be worthwhile to analyze the pattern of cytoplasmic incompatibility among the different *w*Pip groups in Cape Verde and determine whether an island-specific *w*Pip group could be used to regulate *Cx. pipiens* s.l. populations on another island. As an alternative, male *Cx. pipiens* from other regions of the world carrying *w*Pip groups not present in Cape Verde, could be introduced into the archipelago to sterilize local females. It is noteworthy that IIT based on *w*Pip-inducing IC could be a favorable alternative to the costly radiation and genetic manipulation methods, and its implementation would provide a more advantageous solution for low-income nations.

## Conclusion

Our study revealed that *Wolbachia* is widespread in *Cx. pipiens* s.l. from the Cape Verde islands but absent from other mosquito species except for *Cx. tigripes,*, where a novel *Wolbachia* strain was unveiled. The three distinct *w*Pip groups circulating in *Cx. pipiens* s.l. suggest multiple introduction events in the archipelago, possibly of non-African origin. The finding of a novel *Wolbachia* strain in *Cx. tigripes* may provide an additional candidate to be used in biocontrol approaches. Further studies would be required to isolate this new *Wolbachia* strain to be used in transfection studies with major mosquito vectors in order to assess its potential impact on mosquito fitness and vector competence.

## Supplementary Information

**Additional file 1: Table S1. **Primer sequences used for molecular identification of mosquito species collected in Cape Verde islands. **Table S2. **Primers used for PCR detection of *Wolbachia* and genotyping of *w*Pip I–V groups by PCR-RFLP.** Table S3. **Primers used for *Wolbachia *MLST loci and wsp hypervariable region amplification and sequence analysis.

**Additional file 2: Table S4**. Mosquito species collected on each island and tested for *Wolbachia* using *wsp*.

## Data Availability

Sequences generated in this study are available in the GenBank database: *pk1* sequences (OQ223307-OQ223325); *ftsZ* (OQ223326-OQ223348); *hcpA* (OQ223349-OQ223371); *fbpA* (OQ223372-OQ223394); *coxA* (OQ225016-OQ225038); *gatB* (OQ225039-OQ225061); and *wsp* (OQ236526-OQ236548). All reference sequence accession numbers (GenBank) and MLST database IDs are included in the article.

## Abbreviations

CI: Cytoplasmic incompatibility
MLST: Multilocus sequence typing
RFLP: Restriction fragment length polymorphism
Wsp: *Wolbachia* surface protein
