# Supplementary material for: Screening of natural Wolbachia infection in mosquitoes (Diptera: Culicidae) from the Cape Verde islands
Source: Parasit Vectors. 2023 Apr 25;16:142. doi: 10.1186/s13071-023-05745-w (PMC10131387; doi:10.1186/s13071-023-05745-w)
Supplement: Supplementary file 1 — Additional file 1: Table S1. Primer sequences used for molecular identification of mosquito species collected in Cape Verde islands. Table S2. Primers used for PCR detection of Wolbachia and genotyping of wPip I–V groups by PCR-RFLP. Table S3. Primers used for Wolbachia MLST loci and wsp hypervariable region amplification and sequence analysis. [file 13071_2023_5745_MOESM1_ESM.docx]

**Supplementary materials**

Table S1- Primer sequences used for molecular identification of mosquito species collected in Cape Verde islands.

| Species | Primers | Sequences (5’- 3’) | References |
| --- | --- | --- | --- |
| *An. gambiae* complex | AR (*An. arabiensis*) | AAGTGTCCTTCTCCATCCTA | [1] |
|  | ME (*An. melas*) | TGACCAACCCACTCCCTTGA |  |
|  | GA (*An. gambiae*) | CTGGTTTGGTCGGCACGTTT |  |
|  | UN (*Universal*) | GTGTGCCCCTTCCTCGATGT |  |
|  |  |  |  |
| *Culex pipiens* complex | ACEquin | CCTTCTTGAATGGCTGTGGCA | [2] |
|  | ACEpip | GGAAACAACGACGTATGTACT |  |
|  | B1246s | TGGAGCCTCCTCTTCACGG |  |
|  |  |  |  |
| Other species (COI) | LCOI1490_F1 | GGTCAACAAATCATAAAGATATTG | [3] |
|  | HCOI2198_R1 | TAAACTTCAGGGTGACCAAAAAATCA |  |

Table S2- Primers used for PCR detection of Wolbachia and genotyping of wPip I-V groups by PCR-RFLP.

| Target | Primers sequences (5'-3') | Size (bp) | References |
| --- | --- | --- | --- |
| *wsp* | 81F - TGGTCCAATAAGTGATGAAGAAA | 610 | [4] |
|  | 691R - AAAAATTAAACGCTACTCCA |  |  |
|  |  |  |  |
| *Pk1* | pk1 For - CCACTACATTGCGCTATAGA | 1300 | [5] |
|  | pk1 Rev - ACAGTAGAACTACACTCCTCCA |  |  |

Table S3- Primers used for Wolbachia MLST loci and wsp hypervariable region amplification and sequence analysis.

| Target | Primers sequences (5'-3') | Size (bp) | References |
| --- | --- | --- | --- |
| *wsp HVR* | wsp_F1: GTCCAATARSTGATGARGAAAC | 603 | [6,7] |
|  | wsp_R1: CYGCACCAAYAGYRCTRTAAA |  |  |
|  |  |  |  |
| *gatB* | gatB_F1: GAKTTAAAYCGYGCAGGBGTT | 471 |  |
|  | gatB_R1: TGGYAAYTCRGGYAAAGATGA |  |  |
|  |  |  |  |
| *coxA* | coxA_F1: TTGGRGCRATYAACTTTATAG | 487 |  |
|  | coxA_R1: CTAAAGACTTTKACRCCAGT |  |  |
|  |  |  |  |
| *hcpA* | hcpA_F1: GAAATARCAGTTGCTGCAAA | 515 |  |
|  | hcpA_R1: GAAAGTYRAGCAAGYTCTG |  |  |
|  |  |  |  |
| *ftsZ* | ftsZ_F1: ATYATGGARCATATAAARGATAG | 524 |  |
|  | ftsZ_R1: TCRAGYAATGGATTRGATAT |  |  |
|  |  |  |  |
| *fbpA* | fbpA_F1: GCTGCTCCRCTTGGYWTGAT | 509 |  |
|  | fbpA_R1: CCRCCAGARAAAAYYACTATTC |  |  |

**References:**

1. Scott JA, Brogdon WG, Collins FH. Identification of single specimens of the *Anopheles gambiae* complex by the polymerase chain reaction. Am J Trop Med Hyg. 1993;49:520–9.

2. Smith JL, Fonseca DM. Rapid assays for identification of members of the *Culex* (*Culex*) *pipiens* complex, their hybrids, and other sibling species (Diptera: Culicidae). Am J Trop Med Hyg. 2004;70:339-45

3. Folmer O, Black M, Hoeh W, Lutz R, Vrijenhoek R. DNA primers for amplification of mitochondrial cytochrome c oxidase subunit I from diverse metazoan invertebrates. Mol Mar Biol Biotechnol. 1994;3:294–9.

4. Zhou W, Rousset F, O’Neill S. Phylogeny and PCR-based classification of *Wolbachia* strains using wsp gene sequences. Proc R Soc B Biol Sci. 1998;265:509–15.

5. Duron O, Boureux A, Echaubard P, Berthomieu A, Berticat C, Fort P, *et al*. Variability and expression of ankyrin domain genes in *Wolbachia* variants infecting the Mosquito *Culex pipiens*. J Bacteriol. 2007;189:4442.

6. Baldo L, Hotopp JCD, Jolley KA, Bordenstein SR, Biber SA, Choudhury RR, *et al*. Multilocus sequence typing system for the endosymbiont *Wolbachia pipientis*. Appl Environ Microbiol. 2006;72:7098–110.

7. Jolley KA, Bray JE, Maiden MCJ. Open-access bacterial population genomics: BIGSdb software, the PubMLST.org website and their applications. Wellcome Open Res. 2018;3:1–20.
