# Supplementary material for: Screening of natural Wolbachia infection in mosquitoes (Diptera: Culicidae) from the Cape Verde islands
Source: Parasit Vectors. 2023 Apr 25;16:142. doi: 10.1186/s13071-023-05745-w (PMC10131387; doi:10.1186/s13071-023-05745-w)
Supplement: Supplementary file 2 — Additional file 2: Table S4. Mosquito species collected on each island and tested for Wolbachia using wsp. [file 13071_2023_5745_MOESM2_ESM.docx]

Table S4- Mosquito species collected on each island and tested for *Wolbachia* using *wsp*.

| Boavista | | | Brava | | Fogo | | Maio | | Santiago | | Santo Antão | | Total capture (%) | *Wolbachia*  prevalence^a^ |
| --- | --- | --- | --- | --- | --- | --- | --- | --- | --- | --- | --- | --- | --- | --- |
| Adults | | Larvae | Adults | Larvae | Adults | Larvae | Adults | Larvae | Adults | Larvae | Adults | Larvae |  |  |
| *Ae. aegypti* | 6 | 1 | 21 | 11 | 100 | 15 | 66 | 20 | 314 | 67 | 37 | 5 | 663 (40,2%) | 0/663 (0,0%) |
| *Ae. caspius* | - | - | - | - | - | - | 28 | 11 | - | - | - | - | 39 (2,4%) | 0/39 (0,0%) |
| *An. arabiensis* | - | - | - | - | - | - | - | - | 49 | - | - | - | 49 (3,0%) | 0/49 (0%) |
| *An. pretoriensis* | - | - | 40 | 8 | 39 | 5 | - | - | 82 | 9 | 63 | 29 | 275 (16,7%) | 0/275 (0,0%) |
| *C. longioreolata* | - | - | 6 | 8 | - | - | - | - | - | - | 7 | 7 | 28 (1,7%) | 0/28 (0,0%) |
| *Cx. pipiens s.s.* | - | - | - | - | - | - | 6 | - | - | - | 4 | - | 10 (0,6%) | 10/10 (100%) |
| *Cx. quinquefasciatus* | 54 | - | 115 | 41 | 1 | - | 15 | 5 | 247 | 32 | 20 | 15 | 545 (33,1%) | 536/545 (98,3%) |
| *Cx. thalassius* | - | - | - | - | - | - | - | - | 3 | 4 | - | - | 7 (0,4%) | 0/7 (0,0%) |
| *Cx. tigripes* | - | - | - | - | - | - | - | - | 1 | 2 | - | - | 3 (0,2%) | 3/3 (100%) |
| *Hybrids pip/qui* | - | - | - | - | 2 | - | 1 | - | - | - | 18 | 8 | 29 (1,8%) | 29/29 (100%) |

a *Wolbachia* prevalence = number of positive mosquitoes for *wsp*/number tested
